# A comprehensive approach to lifestyle intervention based on a calorie-restricted diet ameliorates liver fat in overweight/obese patients with NAFLD: a multicenter randomized controlled trial in China

**DOI:** 10.1186/s12937-024-00968-8

**Published:** 2024-06-13

**Authors:** Zhong Liu, Piaopiao Jin, Yuping Liu, Zhimian Zhang, Xiangming Wu, Min Weng, Suyan Cao, Yan Wang, Chang Zeng, Rui Yang, Chenbing Liu, Ping Sun, Cuihuan Tian, Nan Li, Qiang Zeng

**Affiliations:** 1 Health Management Center, the First Affiliated Hospital, Zhejiang University School of Medicine, Hangzhou, 310003 China; 2 Department of Health Management, Institute of Health Management, Sichuan Provincial People’s Hospital, University of Electronic Science and Technology of China, Chengdu, 610072 China; 3 Chinese Academy of Sciences Sichuan Translational Medicine Research Hospital, Chengdu, 610072 China; 4 Health Management Center, Qilu Hospital of Shandong University, Jinan, 250012 China; 5 Zhejiang Nutriease Health Technology Company Limited, Hangzhou, 311121 China; 6 Department of Nutrition, The First Affiliated Hospital, Kunming Medical University, Kunming, 650034 China; 7 Health Management Center, Beijing Hospital, National Center of Gerontology, Institute of Geriatric Medicine, Chinese Academy of Medical Sciences, Beijing, 100730 China; 8 Health Management Center, Affiliated Hospital of Qingdao University, Qingdao, 266003 China; 9 Health Management Center, Xiangya Hospital, Central South University, Changsha, 410008 China; 10 Healthcare Center, Union Hospital, Tongji Medical College, Huazhong University of Science and Technology, Wuhan, 430022 China; 11 Health Management Institute, the Second Medical Center & National Clinical Research Center for Geriatric Diseases, Chinese PLA General Hospital, Beijing, 100039 China

**Keywords:** Lifestyle intervention, Fatty liver, Obese, Overweight, Low carbohydrate, High protein.

## Abstract

**Background:**

Nonalcoholic fatty liver disease (NAFLD) is a globally increasing health epidemic. Lifestyle intervention is recommended as the main therapy for NAFLD. However, the optimal approach is still unclear. This study aimed to evaluate the effects of a comprehensive approach of intensive lifestyle intervention (ILI) concerning enhanced control of calorie-restricted diet (CRD), exercise, and personalized nutrition counseling on liver steatosis and extrahepatic metabolic status in Chinese overweight and obese patients with NAFLD.

**Methods:**

This study was a multicenter randomized controlled trial (RCT) conducted across seven hospitals in China. It involved 226 participants with a body mass index (BMI) above 25. These participants were randomly assigned to two groups: the ILI group, which followed a low carbohydrate, high protein CRD combined with exercise and intensive counseling from a dietitian, and a control group, which adhered to a balanced CRD along with exercise and standard counseling. The main measure of the study was the change in the fat attenuation parameter (FAP) from the start of the study to week 12, analyzed within the per-protocol set. Secondary measures included changes in BMI, liver stiffness measurement (LSM), and the improvement of various metabolic indexes. Additionally, predetermined subgroup analyses of the FAP were conducted based on variables like gender, age, BMI, ethnicity, hyperlipidemia, and hypertension.

**Results:**

A total of 167 participants completed the whole study. Compared to the control group, ILI participants achieved a significant reduction in FAP (LS mean difference, 16.07 [95% CI: 8.90–23.25] dB/m) and BMI (LS mean difference, 1.46 [95% CI: 1.09–1.82] kg/m^2^) but not in LSM improvement (LS mean difference, 0.20 [95% CI: -0.19–0.59] kPa). The ILI also substantially improved other secondary outcomes (including ALT, AST, GGT, body fat mass, muscle mass and skeletal muscle mass, triglyceride, fasting blood glucose, fasting insulin, HbA1c, HOMA-IR, HOMA-β, blood pressure, and homocysteine). Further subgroup analyses showed that ILI, rather than control intervention, led to more significant FAP reduction, especially in patients with concurrent hypertension (*p* < 0.001).

**Conclusion:**

In this RCT, a 12-week intensive lifestyle intervention program led to significant improvements in liver steatosis and other metabolic indicators in overweight and obese Chinese patients suffering from nonalcoholic fatty liver disease. Further research is required to confirm the long-term advantages and practicality of this approach.

**Trial registration:**

This clinical trial was registered on ClinicalTrials.gov (registration number: NCT03972631) in June 2019.

**Supplementary Information:**

The online version contains supplementary material available at 10.1186/s12937-024-00968-8.

## Introduction

Nonalcoholic fatty liver disease (NAFLD) is a global health problem that affects approximately one in four people worldwide [1]. NAFLD has become increasingly prevalent in China due to economic development and dramatic lifestyle alterations; it affects an estimated 240 million people, almost one-fifth of the global population with NAFLD [2–4]. The disease poses a tremendous public health burden, as NAFLD is intricately linked to obesity, diabetes, and other metabolic disorders. NAFLD can also lead to cirrhosis and hepatic carcinoma and increase the risk of cardiovascular disease.

Most guidelines and consensus recommend weight loss through lifestyle intervention as the first-line treatment for patients with NAFLD who are overweight and obese [5–9]. However, the ideal diet and physical activity (PA) regimen remains uncertain because previous studies have varied in their outcome measures, the diversity of NAFLD phenotypes, and the lengths of follow-up periods.

A low-calorie diet combined with moderate-intensity PA for an extended period is the current standard lifestyle intervention for NAFLD [9]. This approach has been reported to improve NAFLD and reduce hepatic fat [10, 11]. Recent findings have suggested that a low-carbohydrate and high-protein diet may have a superior effect on NAFLD than a low-fat diet, even with the same calorie restriction [12, 13]. Therefore, it merits investigation to determine whether a low-carbohydrate, high-protein diet is more beneficial than the traditional balanced diet for the Chinese population with NAFLD.

Previous studies indicate that lifestyle intervention for weight loss is not very effective in reality, with fewer than half of participants were able to achieve their preset weight loss goals [14]. This low success rate is influenced by various factors, such as a lack of motivation to adopt a new lifestyle and difficulties in maintaining long-term adherence through self-monitoring [15]. Some studies have suggested that an intensive lifestyle intervention (ILI), incorporating enhanced modification of diet and exercise, frequent counseling with health professionals, and application of novel monitoring techniques, have been identified as promising therapies to achieve successful weight loss and the related therapeutic goals [16–19]. This comprehensive approach has been proven to result in significant weight reduction and reduce the risk of liver fibrosis, type 2 diabetes, and other metabolic diseases in adults with NAFLD with obesity or glucose abnormalities [16, 20–22]. However, most studies have focused on European and American populations, which differ from Asian populations in terms of race, culture, lifestyle, and socioeconomic factors. Additionally, the BMI reference indices for diagnosing obesity and overweight in China differ from those in the West, raising questions about the applicability of the ILI model to Chinese patients with NAFLD.

Currently, no high-quality evidence has elucidated the effect of intensive lifestyle interventions on Chinese patients with NAFLD. A recent multicenter study (ChiCTR1800017463) demonstrated that ILI was effective for both weight management and NAFLD improvement in Chinese adults with obesity [19]. However, the study used the nonalcoholic fatty liver disease score (NFS). This serological formula is less efficient and precise than FibroTouch, FibroScan, or magnetic resonance elastography to evaluate liver steatosis and fibrosis. Therefore, we designed a 12-week multicenter randomized controlled trial (RCT) to examine the effect of a comprehensive ILI approach on overweight and obese patients with NAFLD in the Chinese population. We hypothesized that our comprehensive ILI approach, consisting of a calorie-restricted diet (CRD) with low-carbohydrate and high-protein, exercise, and frequent dietitian-led counseling, could be superior to conventional intervention in improving hepatic steatosis, fibrosis, and other metabolic parameters in overweight and obese patients with NAFLD.

## Materials and methods

### Study design and participant recruitment

This study was a two-arm, multicenter, randomized controlled trial registered on ClinicalTrials.gov (registration number: NCT03972631). Participants were recruited from health management centers and nutrition outpatients of seven Chinese hospitals (the First Affiliated Hospital of Zhejiang University, the Sichuan Provincial People’s Hospital, the Qilu Hospital of Shandong University, the Affiliated Hospital of Qingdao University, Xiangya Hospital of Central South University, the Beijing Hospital, and the First Affiliated Hospital of Kunming Medical University) between August 2019 and April 2021. The study protocol was approved by the Human Ethics and Research Ethics committees of the First Affiliated Hospital of Zhejiang University School of Medicine.

Eligible participants were adults aged 18 to 65 years who had a body mass index (BMI) between 25 and 35 kg/m^2^ and were clinically diagnosed with NAFLD by radiological assessment (ultrasound, computed tomography, or magnetic resonance imaging) [23]. The exclusion criteria are listed in detail in Supplementary Table 1. Before enrollment, all participants were required to sign an informed consent form. At enrollment, demographic data such as age, anthropometry, gender, ethnicity (Han or other), education, marital status, and comorbidities were obtained. Eligible participants were assigned to the ILI or control groups in a 1:1 ratio. This allocation was performed using a computer-generated random sequence stratified by center and gender. Details of the allocation were stored confidential in files on the lifestyle management platform operated by Zhejiang Nutriease Co., Ltd., Hangzhou.

### Lifestyle intervention

During the 12 weeks of the trial, participants in both groups were asked to follow a restricted-energy content (initial weight×25 kcal/kg×0.7). After randomization, participants in the control group were provided with a balanced CRD consisting of 45–55 E% carbohydrates, 20–30 E% fat, and 20–30 E% protein. The dietitian instructed the participants to follow a standard lifestyle intervention based on the Guidelines for the Prevention and Treatment of Nonalcoholic Fatty Liver Disease (2018, China) [24]. Participants in the ILI group were instructed to consume a low-carbohydrate, high-protein diet composed of 20–25% carbohydrate, 30–35% fat, and 40–45% protein per day. All participants were counseled to consume low glycemic index (GI) food as the primary source of carbohydrates, beans, and their products, white meat, and nuts as the main source of protein, and monounsaturated fatty acids as the main source of fat. The principle of ILI was similar to that of the control group, except for the recommendation to choose polyunsaturated fatty acids as the main source of fat. The participants of the ILI group received two nutrition bars (Zhejiang Nutriease Co Ltd, Hangzhou) weighing approximately 112 g per day to replace the staple food of lunch and dinner to help reduce carbohydrate intake while ensuring adequate protein intake. The composition of the bar is revealed in Supplementary Table 2.

Participants in the intervention group received a lifestyle intervention, which included detailed health education, specific diet modification, individualized physical exercise, mobile platform-assisted monitoring, and frequent counseling. The goal of the ILI group was to achieve a weight reduction of 10% of initial body weight. One-on-one health education was held by a dietitian who developed a personal health management plan for every participant based on their stage of obesity. Furthermore, periodic science articles on diet, exercise, and disease hazards were sent through the mobile app (NUTRIEASE 8.9.12, Notte, China) to improve participants’ nutritional knowledge and skills. Except for the three scheduled follow-ups mentioned in the control group (weeks 4, 8, and 12), participants in the ILI group had weekly counseling (lasting at least 15 min) by telephone or app message with a dietitian, who adjusted their diet and PA plans promptly and improved their adherence to the program. Regarding PA, all participants were encouraged to perform at least 150 min of moderate-intensity aerobic exercise per week and resistance exercise twice a week.

### Nutrient and physical assessment

Participants were instructed to record food pictures and meal times 3 days (usually 2 weekdays and 1 weekend day) every four weeks and exercise activities using a mobile phone application (NUTRIEASE 8.9.12, Notte, China) at planned inspection visits. These logs were monitored by researchers and a dedicated dietitian, who conducted online supervision and provided feedback and recommendations using an online lifestyle management system. Dietitians estimated the daily intake of each participant based on the three-day food pictures and the nutrient content according to the nutrient content shown in the Chinese Food Composition Table [25]. Physical activity was assessed using the modified International Physical Activity Questionnaire (long-form) [26], which collected the type, intensity, frequency, and duration of exercise. The weighted metabolic equivalents of task (MET)-min per week (MET·min·wk^–1^) were calculated as duration×frequency per week×MET intensity and then summed across activity domains to produce a weighted estimate of total physical activity from all reported activities per week (MET·min·wk^–1^) [26].

### Study outcomes

The primary outcome was the change in the fat attenuation parameter (FAP), a noninvasive indicator of liver fat content. Key secondary outcomes were the changes in body mass index (BMI) and liver stiffness measurement (LSM). FAP and LSM were measured with iLivTouch (Wuxi HISKY Medical Technologies Co., Ltd.) by an experienced physicist who was blinded to participants’ all clinical data and group assignment, according to the protocol by the manufacturer. The BMI was calculated as body weight in kilograms divided by the square of height in meters. Weight and height were measured after urination and defecation with fasting in the morning.

The other secondary outcomes included changes in systolic and diastolic blood pressure, body composition parameters (body fat mass, muscle mass and skeletal muscle mass), homocysteine, liver function tests (alanine aminotransferase [ALT], aspartate aminotransferase [AST], gamma-glutamyl transferase [GGT]), serum lipids (total cholesterol [TC], low-density lipoprotein cholesterol [LDL-C], high-density lipoprotein cholesterol [HDL-C] and triglycerides [TG]), glucose metabolism biomarkers (fasting blood glucose [FBG], fasting insulin [FINS], glycosylated hemoglobin A1c [HbA1c], Homeostasis Model Assessment of Insulin Resistance [HOMA-IR] and Homeostasis Model Assessment of Beta-cell function [HOMA-β]).

Blood pressure was recorded as the average of two measures while participants were in a seated position after at least 10 min of rest. Body composition was measured while standing on an automated hand-to-foot bioelectric impendence device named the Inbody 770 analyzer (In Body Co., South Korea) with bare feet and light clothing. All subjects strictly followed the instrument’s voice instructions under the supervision of the researchers. Blood samples were collected in the morning after fasting overnight. The Cobas c702 module (Roche Ltd) was used to determine FBG, liver function test, serum lipids, and homocysteine. FINS was assessed using Abbott i2000. HbA1c was detected using Tosoh’s HLC-723G8 automated glycohemoglobin analyzer. HOMA-IR was calculated from fasting plasma glucose and insulin concentrations using the formula: FBG (mmol/L)×FINS (Mu/mL)/22.5 [27, 28]. The HOMA-β was calculated using the formula: 20×FINS (Mu/mL)/[FBG (mmol/L)-3.5] [29]. All outcomes were collected at baseline and 12 weeks after the intervention started, except for BMI, which was additionally measured at 4 and 8 weeks during the intervention.

### Statistical analysis

All analyses were conducted with SPSS version 25.0 (IBM, Armonk, NY, USA) and GraphPad Prism 8.0 (GraphPad Software Inc., San Diego, CA, USA). The primary outcome was the change in FPA between the two groups. The proposed standard deviation (SD) of reduction in FAP was 38 dB/m, according to a preliminary study [30]. Based on differences in treatment effect between groups and a significance level of 0.05, we estimated that an enrollment target of 214 participants (107 per group) would provide the trial with more than 80% statistical power to detect a significant difference of 15 dB/m in FAP between the two interventions. After accounting for an anticipated 20% dropout rate, a total of 257 participants were computed.

The per-protocol analysis set was used to perform all efficacy analyses. No additional imputation methods were applied since the missing data was scarce. Descriptive data were reported as mean ± SD or median (25th, 75th percentile), depending on whether the normal distribution assumptions were met, and categorical data are described with proportions. Differences in the trial outcomes between the two groups were evaluated with the use of the ${\chi }^{2}$ test or Fisher’s test for the categorical variables and the *t*-test or Mann–Whitney U test for the continuous variables. The Shapiro‒Wilk test was used to assess the normality of continuous data. An analysis of covariance (ANCOVA) was performed for primary and secondary outcomes, with treatment as a factor and the baseline value as the continuous covariate. When the assumptions for the ANCOVA were not met, non­parametric ANCOVA was used. Rate difference was calculated with Newcombe method.

The primary outcome was conducted for prespecified subgroup analyses by gender (male vs. female), age category (< 35 vs. ≥ 35 y), BMI category (< 28 vs. ≥ 28 kg/m^2^), ethnicity (Han vs. others), hyperlipidemia (yes vs. no) and hypertension (yes vs. no). We used an interaction term between treatment status and subgroup to assess effect modification by subgroup status.

Grouping differences of the trial outcomes were presented as least-squares (LS) means with 95% confidence intervals (CI). Statistical tests were two-tailed and considered significant when *p* < 0.05 for the primary outcome. To control for type I errors, two key secondary outcomes with Bonferroni correction. The threshold for statistical significance was *p* < 0.025. The *p*-value of other secondary outcomes and subgroup analyses were not adjusted for multiple testing and should be interpreted as exploratory.

## Results

### Study flow and characteristics of the participants at baseline

**Fig. 1:**
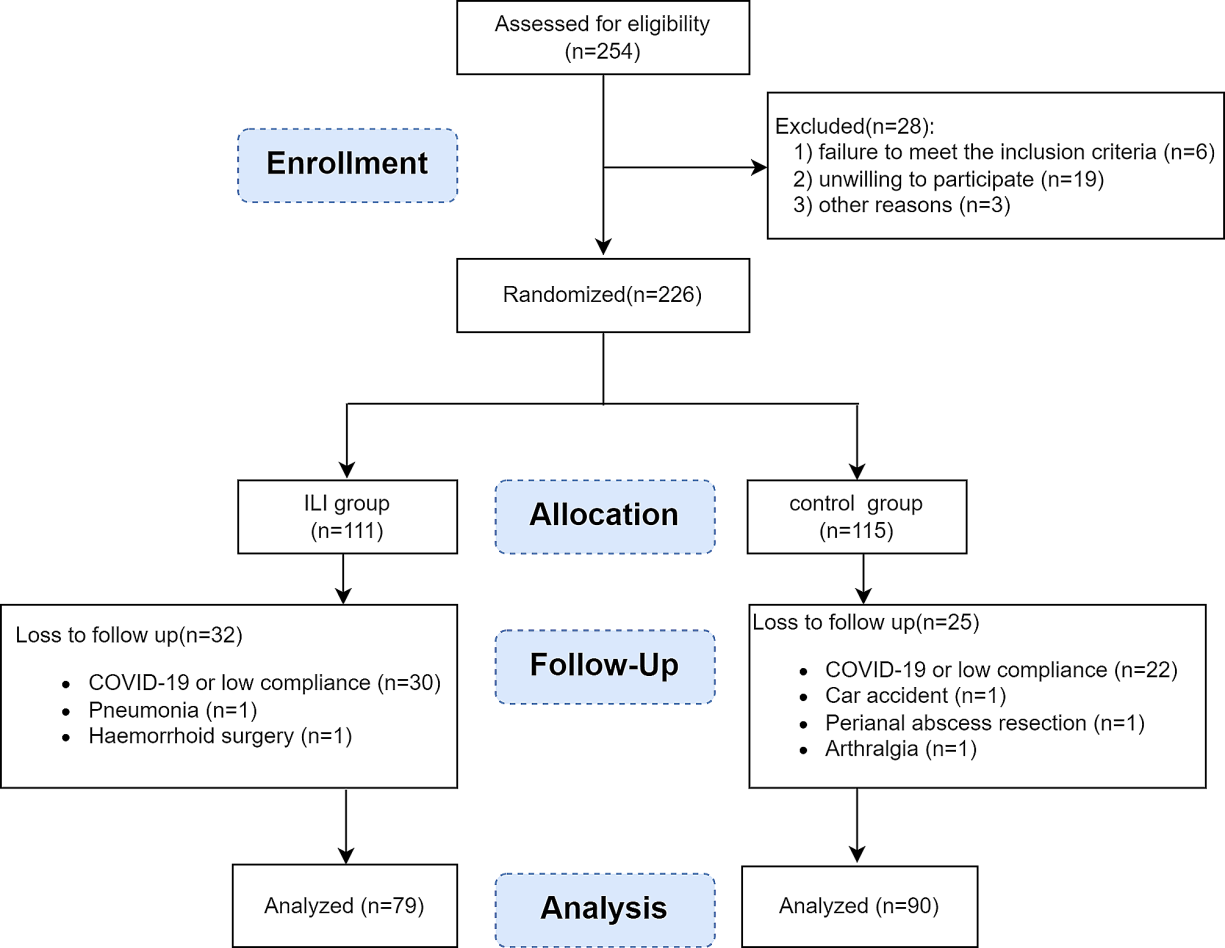
Flow chart of trial participants

The recruitment process included 254 pre-screenings (Fig. 1). Twenty-eight individuals were excluded because of failure to meet the inclusion criteria (*n* = 6), unwillingness to participate (*n* = 19), and other reasons (*n* = 3). A total of 226 participants were included in the initial study and randomly assigned to the control group (*n* = 115) and the ILI group (*n* = 111). Due to the restrictions imposed by the COVID-19 epidemic, recruitment and follow-up became more difficult than expected. Finally, a total of 169 studies were completed (per protocol analysis), and statistical power was secured to evaluate primary and secondary outcomes. Most participants were male (62.7%) and of Han nationality (91.7%), with a mean age of 36.7 (30.8 ~ 41.6) years. The mean BMI was 29.2 (27.1~31.2) kg/m^2^.

The baseline characteristics of the 79 participants in the ILI group and 90 participants in the control group were well-balanced. No significant difference was found in gender, age, anthropometry (e.g., weight, height, and BMI), ethnicity, or marital status between the two groups (Table 1).

**Table 1:** Demographic characteristics of participants at baseline in the per-protocol analysis set

Variable	ILI group (*n* = 79)	Control group (*n* = 90)	*p* value^a^
Gender			
Male, *n* (%)	47 (59.49)	59 (65.56)	0.430
Female, *n* (%)	32 (40.51)	31 (34.44)	
Age, years, median (1st to 3rd quartile)	36.7 (30.55 ~ 40.90)	36.7 (31.1 ~ 42.25)	0.786
Anthropometry			
Height, cm, median (1st to 3rd quartile)	168 (161.25 ~ 174.25)	168 (161.25 ~ 174.75)	0.794
Weight, kg, mean ± SD	83.41 ± 10.21	82.84 ± 13.25	0.750
BMI, kg/m^2^, median (1st to 3rd quartile)	29.57 (27.34 ~ 31.69)	29.15 (27.04 ~ 31.1)	0.676
Ethnicity			
Han, *n* (%)	75 (94.94)	80 (88.89)	0.174
Others, *n* (%)	4 (5.06)	10 (11.11)	
Marital status			
Unmarried, *n* (%)	13 (16.46)	19 (21.11)	0.603
Divorce/separation, *n* (%)	3 (3.8)	2 (2.22)	
Widow, *n* (%)	1 (1.27)	0 (0)	
Married/cohabiting, *n* (%)	62 (78.48)	69 (76.67)	
Education			
Primary school, *n* (%)	1 (1.27)	2 (2.22)	0.476
Junior high school, *n* (%)	3 (3.8)	5 (5.56)	
High school, *n* (%)	1 (1.27)	6 (6.67)	
College and bachelor’s degree n (%)	54 (68.35)	56 (62.22)	
Postgraduate degree, *n* (%)	20 (25.32)	21 (23.33)	
Hyperlipidemia			
Yes, *n* (%)	26 (32.91)	33 (36.67)	0.677
No, *n* (%)	53 (67.09)	57 (63.33)	
Hypertension			
Yes, *n* (%)	9 (11.39)	11 (12.22)	0.999
No, *n* (%)	70 (88.61)	79 (87.78)	

^a^ Fisher’s test was used to compare categorical data between two groups, and the Mann–Whitney U test or the T-test was used to compare the continuous data between two groups

### Effect on the liver outcome

**Fig. 2:**
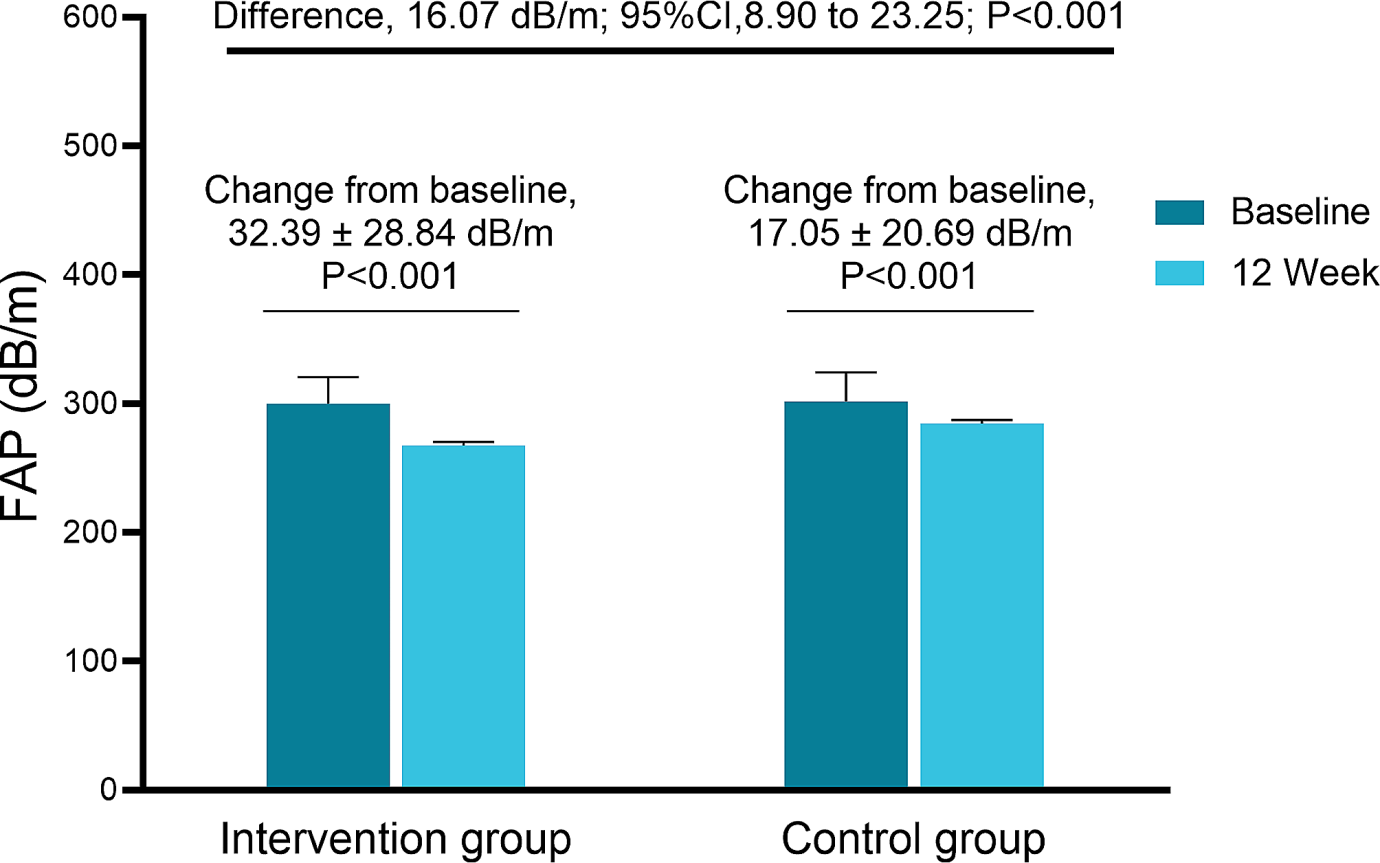
Change in FAP level from baseline to TP12. The change in FAP level was 32.69 dB/m in the intervention group and 17.05 dB/m in the control group. The covariance model showed that the intervention group was significantly better than the control group in reducing the FAP level (LS mean difference: 16.07 [95% CI: 8.90–23.25] dB/m, *p* < 0.001)

After 12 weeks of lifestyle intervention, both groups showed a significant decrease in FAP. The mean FAP level in the control group at week 12 was 284.39 ± 27.31 dB/m, while in the ILI group, it dropped to 267.24 ± 25.56 dB/m (Table 2). The reduction in FAP levels was significantly more significant in the ILI group than in the control group (32.39 ± 28.84 vs. 17.05 ± 20.69 dB/m, *p* < 0.001). The covariance model also confirmed that ILI was more effective than the standard intervention in reducing the FAP level (LS mean difference: 16.07 [95% CI: 8.90–23.25] dB/m, *p* < 0.001), which implied that the ILI was superior to the standard intervention in reducing FAP levels in NAFLD (Fig. 2).

**Table 2:** Change in primary and secondary outcomes from baseline to 12 weeks after enrollment using a protocol analysis

Outcome variable	Time	ILI (*n* = 79)	Control (*n* = 90)	Difference of change^a^ (95% CI)	*p*_−_value
Liver Fibrotouch					
FAP (dB/m)	Baseline	299.88 ± 20.80	301.56 ± 22.72	16.07 (8.90–23.25)	< 0.001
	12 weeks	267.24 ± 25.56^b^	284.39 ± 27.31^b^		
LSM (kPa)	Baseline	6.74 ± 1.98	6.56 ± 1.62	-0.20 (-0.19-0.59)	0.317
	12 weeks	5.58 ± 1.22^b^	5.79 ± 1.42^b^		
Anthropometry					
BMI (kg/m^2^)	Baseline	29.37 ± 2.59	29.23 ± 2.65	1.46 (1.09–1.82)	< 0.001
	12 weeks	26.69 ± 2.41^b^	28.01 ± 2.76^b^		
Body composition					
Body fat mass (kg)	Baseline	29.29 ± 5.99	28.73 ± 6.43	3.33 (2.48–4.19)	<0.001
	12 weeks	23.01 ± 5.61^b^	25.70 ± 6.25^b^		
Muscle mass (kg)	Baseline	51.09 ± 8.12	50.84 ± 9.54	0.97 (0.26–1.68)	0.008
	12 weeks	49.75 ± 7.89^b^	50.40 ± 8.85^b^		
Skeletal muscle mass (kg)	Baseline	30.30 ± 5.25	30.30 ± 5.93	0.45(0.12–0.78)	0.007
	12 weeks	29.49 ± 5.11^b^	29.88 ± 5.66		
Blood pressure monitoring					
SBP (mmHg)	Baseline	126.99 ± 12.88	123.03 ± 11.80	3.41 (0.81-6.00)	0.011
	12 weeks	119.16 ± 9.86^b^	120.45 ± 11.5^b^		
DBP (mmHg)	Baseline	80.84 ± 9.77	78.39 ± 8.06	2.83 (0.77–4.90)	0.008
	12 weeks	75.49 ± 7.37^b^	76.98 ± 8.93		
Liver function test					
ALT (U/L)	Baseline	43.43 ± 26.59	39.87 ± 23.20	4.06 (1.12–7.01)	0.007
	12 weeks	21.66 ± 10.07^b^	24.56 ± 11.11^b^		
AST (U/L)	Baseline	28.44 ± 11.18	26.47 ± 9.69	2.20 (0.21–4.22)	0.030
	12 weeks	20.05 ± 6.09^b^	21.82 ± 7.74^b^		
GGT (U/L)	Baseline	39.03 ± 21.44	36.59 ± 19.18	7.82 (5.17–10.47)	<0.001
	12 weeks	20.76 ± 11.87^b^	26.69 ± 11.45^b^		
Glucose metabolism biomarkers					
FPG (mmol/L)	Baseline	5.25 ± 0.55	5.29 ± 0.50	0.09 (-0.05-0.20)	0.209
	12 weeks	5.10 ± 0.51^b^	5.21 ± 0.50		
FINS (mIU/L)	Baseline	12.68 ± 6.37	13.19 ± 6.13	1.98 (0.87–3.10)	0.001
	12 weeks	8.25 ± 3.84^b^	10.25 ± 4.41^b^		
HbA1c (%)	Baseline	5.56 ± 0.39	5.54 ± 0.36	0.15 (0.07–0.23)	< 0.001
	12 weeks	5.35 ± 0.37^b^	5.50 ± 0.35		
HOMA-IR	Baseline	3.02 ± 1.65	3.19 ± 1.67	0.45 (0.16–0.73)	0.003
	12 weeks	1.92 ± 0.97^b^	2.39 ± 1.10^b^		
HOMA-β	Baseline	147.46 ± 76.22	158.69 ± 82.45	24.12 (9.72–38.50)	0.001
	12 weeks	104.52 ± 46.42^b^	129.55 ± 62.50^b^		
Lipid panel screen					
TC (mmol/l)	Baseline	4.91 ± 0.90	4.89 ± 0.76	0.09 (-0.26-0.09)	0.330
	12 weeks	4.85 ± 0.88	4.71 ± 0.75^b^		
LDL-C (mmol/L)	Baseline	3.04 ± 0.72	3.04 ± 0.70	0.03 (-0.17-0.12)	0.725
	12 weeks	2.98 ± 0.69	2.96 ± 0.67		
HDL-C (mmol/L)	Baseline	1.18 ± 0.27	1.16 ± 0.22	0.03 (-0.08-0.21)	0.239
	12 weeks	1.22 ± 0.24	1.18 ± 0.24		
TG (mmol/L)	Baseline	2.04 ± 1.02	1.94 ± 1.01	0.27 (0.11–0.42)	0.001
	12 weeks	1.17 ± 0.59^b^	1.39 ± 0.62^b^		
Hcy (µmol/L)	Baseline	11.85 ± 4.77	11.47 ± 4.23	0.98 (0.29–1.66)	0.006
	12 weeks	10.00 ± 3.06^b^	10.46 ± 2.27		

^a^Least-squares (LS) means the difference in change between two groups. ^b^*p* < 0.05 between baseline and 12 weeks

**Fig. 3:**
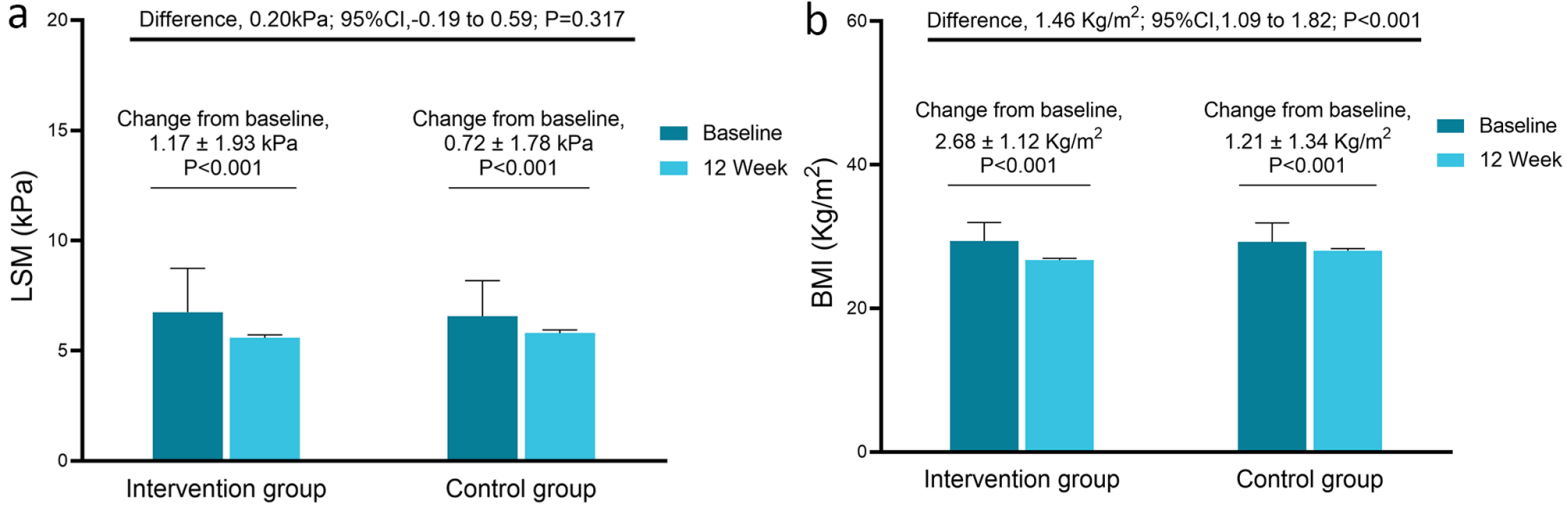
Change in LSM and BMI from baseline to TP12. **(a)** Change in LSM from baseline to TP12. The ILI group had a more significant decrease in LSM than the control group (1.17 ± 1.93 kPa vs. 0.72 ± 1.78 kPa, respectively). The covariance model showed no significant difference between the two groups in reducing the LSM level (LS mean difference: 0.20 [95% CI: -0.19-0.59] kPa, *p* = 0.317). **(b)** Change in BMI from baseline to TP12. The ILI group had a more significant decrease in BMI than the control group (2.68 ± 1.12 kg/m^2^ vs. 1.21 ± 1.34 kg/m^2^, respectively). The covariance model showed that the ILI group was significantly better than the control group in reducing the BMI level (LS mean difference: 1.46 [95% CI, -1.09-1.82] kg/m^2^, *p* < 0.001)

LSM was significantly decreased by 1.17 ± 1.93 kPa in the ILI group and 0.72 ± 1.78 kPa in the control group (*p* < 0.001, Fig. 3a). There were no significant differences between the two groups in improving liver fibrosis (LS mean difference: 0.20 [95% CI: -0.19-0.59] kPa, *p* = 0.317).

Liver enzymes, including ALT, AST, and GGT, were all significantly reduced in both groups after 12 weeks of intervention (*p* < 0.05, Table 2). The reduction of ALT, AST and GGT in the ILI group was more significant than that in the control group (LS mean difference_ALT_: 4.06 [95% CI: 1.12–7.01]U/L, *p*_ALT_=0.007; LS mean difference_AST_:2.20 [95% CI: 0.21–4.22]U/L, *p*_AST_=0.030 and LS mean difference_GGT_: 7.82 [95% CI, 5.17–10.47]U/L, *p*_*GGT*_<0.001).

### Effect on anthropometry and body composition

As indicated in Fig. 3b, the ILI group had a significantly higher reduction in BMI than the control group (2.68 vs. 1.21 kg/m^2^, LS mean difference 1.46 [95% CI: -1.09-1.82] kg/m^2^, *p* < 0.001). The proportion of participants who achieved the goal of BMI reduction (≥ 10%) was 40.5% (32/79) in the ILI group and 10.1% (9/89) in the control group (difference of change: 30.4% [95% CI: 16.6 – 43.1%], *p* < 0.001). Body composition analysis also revealed that both groups experienced a reduction in body fat mass and muscle mass after 12 weeks of intervention (*p* < 0.05). However, only the ILI group had a reduction in skeletal muscle mass (*p* < 0.05).

### Effect on lipid metabolism and insulin resistance

TG decreased in both groups but to a significantly larger extent in the ILI group than in the control group (LS mean difference: 0.27 [95% CI: 0.11–0.42] mmol/L, *p* = 0.001). The two groups showed no significant difference in TC, LDL-C, and HDL-C. However, in the control group, TC improved significantly after 12 weeks of intervention (*p* < 0.05). Fasting insulin, HbA1c, HOMA-IR, and HOMA-β were all decreased in both groups, and the reductions were significantly more significant in the ILI group than in the control group (*p* < 0.05). However, no significant change was found between groups in lowering the FBG (LS mean difference: 0.09 [95% CI: -0.05-0.20] mmol/L, *p* = 0.209). Fasting blood glucose decreased significantly from baseline to week 12 in the ILI group (*p* < 0.05), while the control group did not have a significant change.

### Effect on cardiovascular risk factors

The effects of ILI on blood pressure and homocysteine are displayed in Table 2. Compared to the control group, the decrease in SBP and DBP was significantly higher in the ILI group (LS mean difference SBP: 3.41 [95% CI: 0.81–6.00] mmHg, *p*_SBP_ =0.011 and LS mean difference DBP: 2.83 [95% CI: 0.77–4.90] mmHg, *p*_DBP_ =0.008). After 12 weeks of treatment, SBP and DBP in the ILI group were significantly lower than the baseline values (*p* < 0.05). In contrast, the control group only significantly improved SBP (*p* < 0.05).

Homocysteine (Hcy) is recognized as a potential predictor of subclinical atherosclerosis and correlates with cardiovascular disease prevalence [31]. ILI was more effective than the conventional intervention in lowering Hcy levels (LS mean difference: 0.98 [95% CI: 0.29–1.66] µmol/L, *p =* 0.006]. Furthermore, only the ILI group had a significant decrease in Hcy levels from baseline (10.00 ± 3.06 vs. 11.85 ± 4.77 µmol/L, *p* < 0.05).

### Assessment of dietary intake and physical activity

At baseline, there were no significant differences in total energy intake, dietary intake, or physical activity (Table 3). At the end of the study, both groups significantly reduced total energy and carbohydrate intake. However, members of the ILI group significantly increased protein and fiber intake (Table 3). Fat intake remained unchanged between baseline and week 12 in both groups. An increase in energy from protein and fat was also observed at week 12, with a significant difference between the two groups. At 12 weeks, physical activity improved significantly within the ILI group (98.4 ± 8.1vs. 69.3 ± 80.3 MET-h/week, *p* < 0.05) but did not differ between the two groups or within the control group.

**Table 3:** Analysis of dietary intake and physical activity at baseline and post-intervention

Variables	Time	ILI (*n* = 79)	Control (*n* = 90)	*p*_−_value
Total energy intake (kcal)				
	Baseline	1650 ± 658	1578 ± 574	0.449
	12 weeks	1288 ± 299^b^	1377 ± 497^b^	0.149
Energy from carbohydrate (%)				
	Baseline	42.4 ± 11.9	44.1 ± 10.4	0.074
	12 weeks	24.6 ± 8.5^b^	39.1 ± 11.0 ^b^	< 0.001
Energy from fat (%)				
	Baseline	39.1 ± 9.3	37.8 ± 8.8	0.13
	12 weeks	45.2 ± 7.7 ^b^	39.9 ± 10.4 ^b^	< 0.001
Energy from protein (%)				
	Baseline	19.0 ± 5.2	18.8 ± 5.2	0.657
	12 weeks	32.5 ± 6.9^b^	21.5 ± 6.5^b^	< 0.001
Carbohydrate intake (g)				
	Baseline	174.9 ± 86.7	175.1 ± 77.1	0.511
	12 weeks	77.6 ± 29.6^b^	133.5 ± 59.5 ^b^	< 0.001
Fat intake (g)				
	Baseline	72.1 ± 35.5	66.2 ± 29.6	0.163
	12 weeks	65.3 ± 21.3	61.8 ± 35.1	0.001
Protein intake (g)				
	Baseline	78.0 ± 36.9	74.0 ± 33.4	0.353
	12 weeks	104.3 ± 31.4^b^	74.0 ± 33.6	< 0.001
Dietary fiber intake (g)				
	Baseline	10.7 ± 9.4	9.5 ± 6.7	0.16
	12 weeks	13.1 ± 7.4^b^	8.6 ± 8.8^b^	< 0.001
Physical activity (MET-h/week)				
	Baseline	69.3 ± 80.3	70.6 ± 77.6	0.679
	12 weeks	98.4 ± 8.1^b^	74.2 ± 81.9	0.218

^b^ significantly different compared to the baseline (*p* < 0.05)

### Subgroup analysis

**Fig. 4:**
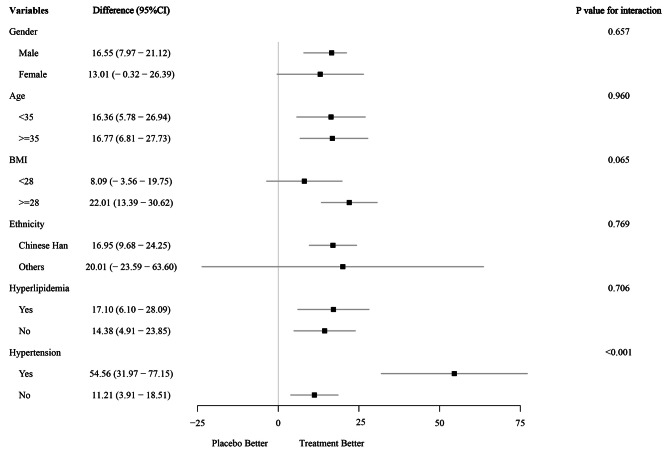
Prespecified subgroup analysis based on gender, age, ethnicity, hyperlipidemia, and hypertension

We performed a prespecified subgroup analysis to examine whether demographic factors influenced the effect of the intervention on FAP (Fig. 4). We stratified the participants by gender (male/female), age (< 35/ ≥35 y), BMI (< 28/ ≥28 kg/m^2^), ethnicity (Han Chinese/others), hyperlipidemia (yes/no), and hypertension (yes/no). The results showed that there was no significant treatment heterogeneity in FAP reduction between the two intervention groups by age, gender, ethnicity, and hyperlipidemia, except hypertension (*p*-value for interaction < 0.001). However, within the ILI group, the participants who were male (LS mean difference: 16.55 [95% CI: 7.97–21.12] dB/m), had BMI ≥ 28 kg/m^2^ (LS mean difference: 22.01 [95% CI: 13.39–30.62] dB/m) or belonged to Han Chinese (LS mean difference: 16.95 [95% CI: 9.68–77.15] dB/m) had a more significant decrease in FAP than their counterparts.

### Adverse events

During 12 weeks of intervention, the most common adverse events were gastrointestinal symptoms, such as constipation (two participants in the ILI group and one in the control group) and diarrhea (two participants in the ILI group). Four participants had upper respiratory tract infections (three in the ILI group and one in the control group). Four participants suffered joint sprains during exercise (two in the ILI group and two in the control group). In the ILI group, there were also reports of fatigue, pneumonia, lymphadenitis, and serum creatinine (one each). In the control group, there were also reports of knee pain, depression, and a car accident (one, two, and one, respectively). The other adverse events unrelated to the interventions are presented in Supplementary Table 3.

## Discussion

In this multicenter randomized controlled trial, both lifestyle intervention strategies significantly decreased FAP, LSM, and liver enzymes in patients with NAFLD. However, our comprehensive ILI approach produced more substantial reductions in FAP and liver enzymes compared to the traditional interventions described in the current guidelines. Furthermore, the ILI approach markedly improved extrahepatic parameters, including BMI, body composition, blood pressure, homocysteine levels, triglycerides, and insulin resistance. These findings indicate that our ILI method is more effective in managing weight and improving metabolic health in overweight or obese NAFLD patients in China.

In our study, specific diet modifications played an essential role in the intensive lifestyle intervention. We found that a CRD with low carbohydrate and high protein, compared with the balanced diet with the same energy limitation used in the conventional intervention, was more effective in NAFLD management. A calorie reduction of 500 to 1000 kcal per day in a CRD regimen, as recommended by most guidelines, can improve intra- and extrahepatic metabolism by decreasing insulin resistance, improving liver inflammation, and altering gut microbiota [23]. Notably, the total calorie reduction and the optimal composition of macronutrients (carbohydrate, fat, and protein) in a diet design contributed to the metabolic outcomes. Specifically, low-carbohydrate diets have shown remarkable therapeutic efficacy in metabolic regulation and thus have been increasingly applied in treating obesity, NAFLD, and other metabolism-related diseases. Based on the advantages of low-carbohydrate diets in increasing energy expenditure and decreasing body weight observed in many preclinical studies [32], a low-carbohydrate diet has been validated to be more effective in reducing the intrahepatic fat content than a simple hypocaloric diet [33, 34]. The underlying mechanisms involved reduced hepatic *de novo* lipogenesis (DNL) by restricting glucose or fructose intake, which led to decreased synthesis of fatty acids and accumulation of intra-liver fat [35]. Another underlying mechanism was the improved insulin resistance by low carbohydrate diets, which enhanced lipolysis and reduced fatty acids delivered to the liver [36]. In the context of calorie restrcition, low-carbondydrate diets also have been regarded to have stronger impact on NAFLD outcomes in comparison with fat intake control [12, 34], which helps to explain the scenarios in our study that increasing fat intake in the low-carbonhydrate settings in the ILI group did not alter the final results, especially serum lipid level.

Low-carbohydrate and high-protein diets have been shown to reduce liver fat, improve glucose homeostasis, and promote weight loss in patients with metabolic syndrome and NAFLD [12]. Compared to normal or low-protein diets, high-protein diets significantly reduced liver fat content in several studies [13, 37, 38], as confirmed by our study. Further studies also suggested that high-protein diets could prevent and reverse hepatic steatosis development, regardless of dietary carbohydrate, fat, or overall caloric intake [39, 40]. High-protein diets have additional benefits for NAFLD outcomes. However, excessive protein intake can also increase serum creatine levels, as observed in one participant during the study period, indicating that renal function should be monitored in susceptible individuals.

We also found that intensive counseling enhanced the efficacy of ILI in NAFLD treatment. However, it is challenging to motivate individuals to adopt and maintain an intervened lifestyle [41], as they may face barriers such as limited food choice, exercise-induced pain and fatigue, unhealthy food cravings, and delayed benefits. Previous studies have disclosed that frequent professional counseling on medical nutrition therapy improves the outcomes of simple diet and exercise interventions [20, 42–46]. Therefore, we applied this strategy in our ILI approach, where the dietitians used a motivational interview with a four-step protocol (engaging, focusing, evoking, and planning) to facilitate behavioral change [41]. Notably, our intensive counseling consisted of weekly one-to-one communication supported by a smartphone app that allowed dietitians to provide timely feedback on patients’ diets and physical activity based on detailed records uploaded from mobile devices [18]. These strategies reinforced the self-monitoring behavior that led to sustained weight loss. Furthermore, as a positive result acquired in a short time, rapid weight loss in a short time could act as an incentive to increase adherence to the intervention.

Our findings also demonstrated that weight loss was a key factor in NAFLD resolution. In our study, ILIs reduced BMI by 9%, twice as much as the control group (approximately 4%). This indicated that ILIs successfully achieved the weight control goal (defined as a 7–10% reduction in initial weight) [5]. Previous clinical trials have shown that 5% weight loss after lifestyle changes improved steatosis reversion [22], normalized liver enzymes [47], and reduced the risk of diabetes and other metabolic disorders [48]. However, our results demonstrated that even a 4% BMI reduction in the control group improved multiple liver parameters, suggesting that any degree of weight control would eventually benefit NAFLD treatment. Moreover, our analyses revealed a more significant reduction in FAP and transaminase levels in the ILI group than in the control group, indicating that the beneficial effects on clinical and histological outcomes were closely associated with the degree of BMI reduction in a dose-dependent manner.

In addition to body weight, body fat distribution is another indicator of metabolic status in NAFLD, as hepatic fat content and its related metabolic parameters could decrease in NAFLD patients without noticeable changes in body weight change [49]. Therefore, analyzing body composition, especially body fat mass, is an important way to evaluate the effect of dietary intervention [50]. Our data showed a significant reduction in body fat mass in the ILI group, confirming the superior effect of ILIs on NAFLD-related metabolic disorders. Notably, ILIs also reduced muscle mass and skeletal muscle mass. This may be due to decreased muscle protein synthesis and increased muscle proteolysis caused by CRD during calorie restriction [51–53]. To prevent weight-induced muscle mass loss, resistance exercise training, and high protein intake should be applied [54–56].

Compared to conventional treatment, our comprehensive approach to ILI improves specific cardiovascular markers, such as homocysteine and systolic and diastolic blood pressure. This may be due to the low carbohydrate content, which has been proven to lower blood pressure and reduce estimated cardiovascular risk in the “Omni Heart Randomized Trial” [57]. Moreover, reducing BMI is crucial for patients with hypertension, as 1 kg of body weight loss leads to a 1 mmHg BP reduction, according to the latest guidelines for essential hypertension [58]. Interestingly, our further prespecified subgroup analysis showed that the effect of ILI on reducing intrahepatic fat was more pronounced in patients with prehypertension than in those without hypertension. There is some evidence that prehypertension is associated with the incidence and progression of NAFLD. A meta-analysis of 11 cohort studies in patients with NAFLD, confirmed by liver biopsies, showed that hypertension (HTN) at baseline almost doubled the risk of progression of fibrosis progression [59]. The activated renin-angiotensin system (RAS) in HTN may impair hepatic function by increasing oxidative stress, apoptosis, and inflammation [60]. Therefore, we speculated that the improvement in HTN could assist in inhibiting the synthesis of hepatic fat in patients with NAFLD by regulating the RAS pathway. However, the population with pre-HTN in our study was small. More studies are required to confirm this conclusion and clarify the underlying mechanism.

The strength of this article lies in one-on-one nutritional counseling coupled with a three-month follow-up, which can improve patient compliance. This mainly benefits the Chinese population, where nutritional counseling is not as common. Additionally, this study provides an analysis of dietary components, which are relatively rare among the Chinese population due to the diverse and complex nature of Chinese cuisine, making nutrient calculations challenging. We use standardized dietary software and nutritionist training to calculate the participants’ nutrient intake. However, there are some limitations in the present RCT. First, the dropout rate during follow-up was 26%, which could be impacted by unexpected quarantine policies during the COVID-19 pandemic and could potentially compromise the results of this study. Due to the pandemic-related loss of follow-up, we could not accurately perform the intention-to-treat analyses since the final status of the participants lost to follow-up was primarily unclear. Second, the duration of the intervention was only 12 weeks, during which the patients were closely monitored. Hence, the long-term benefits after the intervention period are uncertain, especially for those patients with NAFLD who returned to the normal diet after the trial. A further follow-up study should be performed to predict the long-term risk of steatosis recurrence after the intervention. Finally, our study lacks precise characterization of some variables, such as the intensity of physical activity, the intake amount of different types of unsaturated fatty acid, the histological assessment of steatosis, the unavailability of more established tools for subclinical atherosclerosis diagnosis (such as brachial-ankle pulse wave velocity in addition to the Hcy level alone), which all might introduce bias to the final results.

## Conclusion

In conclusion, this 12-week multicenter randomized controlled trial demonstrated that ILIs, comprising a low-carbohydrate, high-protein calorie-restricted diet along with exercise, significantly enhanced liver steatosis and other metabolic parameters in overweight Chinese patients with NAFLD. Compared to standard interventions, the provision of diet and exercise therapy by registered dietitians at a high frequency appears to be more effective. Further research is needed to explore the long-term benefits and practicality of ILIs in real-world settings.

### Electronic supplementary material

Below is the link to the electronic supplementary material.

Supplementary Material 1

## Data Availability

The data presented in this study are available upon request from the corresponding author. The data are publicly unavailable because individual privacy may be compromised.

## Abbreviations

NAFLD: Nonalcoholic fatty liver disease
ILI: Intensive lifestyle intervention
FAP: Fat attenuation parameter
BMI: Body index mass
CRD: Calorie-restricted diet
LSM: Liver stiffness measurement
PA: Physical activity
RCT: Randomized controlled trial
GI: Glycemic index
MET·min·wk^–1^: (MET)-minutes per week
ALT: Alanine aminotransferase
AST: Aspartate aminotransferase
GGT: Gamma-glutamyl transferase
TC: Total cholesterol
LDL-C: Low-density lipoprotein cholesterol
HDL-C: High-density lipoprotein cholesterol
TG: Triglycerides
FBG: Fasting blood glucose
FINS: Fasting insulin
HbA1c: Glycosylated hemoglobin A1c
HOMA-IR: Homeostasis Model Assessment of Insulin Resistance
HOMA-β: Homeostasis Model Assessment of Beta-cell Function
SD: Standard deviation
LS: Least-squares
CIs: Confidence intervals
Hcy: Homocysteine
DNL: De novo lipogenesis
HTN: Hypertension
RAS: Renin-angiotensin system
