# Supplementary material for: A comprehensive approach to lifestyle intervention based on a calorie-restricted diet ameliorates liver fat in overweight/obese patients with NAFLD: a multicenter randomized controlled trial in China
Source: Nutr J. 2024 Jun 13;23:64. doi: 10.1186/s12937-024-00968-8 (PMC11170812; doi:10.1186/s12937-024-00968-8)
Supplement: Supplementary file 1 — Supplementary Material 1 [file 12937_2024_968_MOESM1_ESM.docx]

**A comprehensive approach to lifestyle intervention based on a calorie-restricted diet ameliorates liver fat in overweight/obese patients with NAFLD: A multicenter randomized controlled trial in China**

**Supplementary Materials**

Zhong Liu^1,†^, Piaopiao Jin^1,†^, Yuping Liu^2,3^, Zhimian Zhang^4^, Xiangming Wu^5^, Min Weng^6^, Suyan Cao^7^, Yan Wang^8^, Chang Zeng^9^, Rui Yang^10^, Chenbing Liu^1^, Ping Sun^2,3^, Cuihuan Tian^4^, Nan Li^1^, Qiang Zeng^11,*^

† These authors contributed equally to this work.

1. Health Management Center, the First Affiliated Hospital, Zhejiang University School of Medicine, Hangzhou 310003, China

2. Department of Health Management and Institute of Health Management, Sichuan Provincial People's Hospital, University of Electronic Science and Technology of China, Chengdu 610072, China

3. Chinese Academy of Sciences Sichuan Translational Medicine Research Hospital, Chengdu 610072, China

4. Health Management Center, Qilu Hospital of Shandong University, Jinan 250012, China

5. Zhejiang Nutriease Health Technology Company Limited, Hangzhou 311121, China

6. Department of Nutrition, The First Affiliated Hospital, Kunming Medical University, Kunming 650034, China

7. Health Management Center, Beijing Hospital, National Center of Gerontology, Institute of Geriatric Medicine, Chinese Academy of Medical Sciences, Beijing 100730, China

8. Health Management Center, Affiliated Hospital of Qingdao University, Qingdao 266003, China

9. Health Management Center, Xiangya Hospital, Central South University, Changsha, 410008, China

10. Healthcare Center, Union Hospital, Tongji Medical College, Huazhong University of Science and Technology, Wuhan 430022, China

11. Health Management Institute, the Second Medical Center & National Clinical Research Center for Geriatric Diseases, Chinese PLA General Hospital, Beijing 100039, China

* Corresponding author. Email: zq301@126.com.

**Supplementary Table 1**

| **Exclusion criteria** |
| --- |
| - Patients with a history of excessive alcohol consumption (≥ 210 g/week for males and ≥ 140 g/week for females in the past 12 months). - Patients with acute or chronic viral hepatitis, autoimmune hepatitis, cirrhosis, liver cancer, or severe liver function damage defined by liver enzyme levels exceeding 3 times the normal upper limit. - Patients with specific diseases that can lead to the occurrence of fatty liver (total parenteral nutrition and celiac disease). - Patients with the use of hepatoprotective drugs within three months. - Patients with the use of medications known to cause liver damage (acetoiofurone, methotrexate, tamoxifen, glucocorticoids, and estrogen). - Patients with the use of antihistamines, beta-blockers, calcium antagonists, contraceptives, and other drugs that may affect weight. - Patients with renal dysfunction or other kidney diseases that require the control of protein intake. - Patients with diseases that affect food digestion and absorption, such as chronic diarrhea, constipation, severe digestive tract inflammation, active peptic ulcer, post gastrointestinal surgery, and cholecystitis/postcholecystectomy) - Patients with a history of diabetes, prediabetes, serious cardiovascular and cerebrovascular diseases, uncontrolled severe hypertension, anemia, and cancer. - Patients with mental disorders, memory disorders, epilepsy, or with the use of antiepileptic treatment, anti-schizophrenia, and antidepressants. - Patients with secondary obesity caused by endocrinological, genetic, and central nervous system disorders. - Patients suffering from infectious diseases such as tuberculosis and AIDS. - Pregnant women or breastfeeding women. - Patients with physical disability. - Patients with massive ascites or right epigastric wounds that affect the examination of fibrotouch. |

**Supplementary Table 2. Composition Information of the Nutrition Bar.**

| Items | Per 100 g | Nutrient Reference Value% |
| --- | --- | --- |
| Energy | 1418kJ | 17% |
| Protein | 31.2 g | 52% |
| Fat | 14.3 g | 24% |
| Trans Fatty Acids | 0 g |  |
| Carbohydrate | 28.6 g | 10% |
| Dietary Fiber | 4.9 g | 20% |
| Sodium | 347 mg | 17% |

**Supplementary Table 3. Adverse events during the 12-week intervention.**

| Adverse event | ILI  (n=111) | Control  (n=115) | Total participants (n=226) |
| --- | --- | --- | --- |
| Total | 15 (16%) | 12 (10%) | 27 (12%) |
| Constipation | 2 (2%) | 1 (1%) | 3 (1%) |
| Diarrhea | 2 (2%) | 0 (0%) | 2 (1%) |
| Upper respiratory tract Infection ( | 3 (3%) | 1 (1%) | 4 (2%) |
| Sprain | 2 (2%) | 2 (2%) | 4 (2%) |
| Depression | 0 (0%) | 2 (2%) | 2 (1%) |
| Fatigue | 1 (1%) | 0 (0%) | 1 (0%) |
| Pneumonia | 1 (1%) | 0 (0%) | 1 (0%) |
| Chronic appendicitis | 1 (1%) | 0 (0%) | 1 (0%) |
| Periodontitis | 0 (0%) | 1 (1%) | 1 (0%) |
| Pharyngitis | 0 (0%) | 1 (1%) | 1 (0%) |
| Knee pain | 0 (0%) | 1 (1%) | 1 (0%) |
| Cervical lymphadenitis | 1 (1%) | 0 (0%) | 1 (0%) |
| Hemorrhoids | 1 (1%) | 0 (0%) | 1 (0%) |
| Perianal abscess resection | 0 (0%) | 1 (1%) | 1 (0%) |
| Shingles | 0 (0%) | 1 (1%) | 1 (0%) |
| Hyperuricemia | 1 (1%) | 0 (0%) | 1 (0%) |
| Car Accident | 0 (0%) | 1 (1%) | 1 (0%) |
